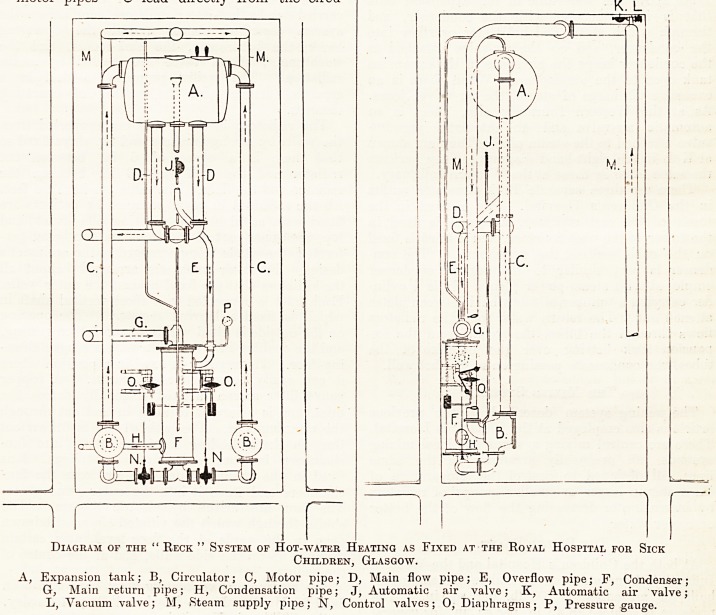# The Royal Hospital for Sick Children, Glasgow

**Published:** 1915-10-02

**Authors:** 


### THE HEATING OF HOSPITALS.*

**[NOTE.—In a series of articles the writer proposes to describe the different systems of heating that are employed in hospitals. He will be pleased to answer any questions through "The Hospital," bearing upon the subject of heating or ventilation. Each system will be illustrated and described, as exemplified at some hospital where it has been applied. In the first article the method by the use of low-pressure steam employed at Leicester Infirmary was dealt with.]**

### III. The Royal Hospital for Sick Children, Glasgow.

### THE LATER FORM OF THE "RECK" SYSTEM.

In the Children's Hospital at Glasgow a later arrangement and, as the writer thinks, an improved one, of the Reck system has been fixed. It is shown diagrammatically in fig. 1. As will be seen from the diagram, there are two circulators but one expansion tank and one condenser. The "motor pipes" C lead directly from the circulators to the upper part of the expansion tank, one on each side. The steam pipe M is taken up to a point above the expansion tank, as at the Western Infirmary, and two branch pipes are taken from it, one on each side, to the circulators. There are two flow mains, marked D on the diagram, both leading from the bottom of the expansion tank. There is one return main, marked G on the diagram, leading to the condenser, the two circulators being connected to the pipes leading from the other side of the condenser. The overflow pipe is shown in the expansion tank; it leads right down to the condenser; the steam, which, it is claimed, separates from the motor column entering from the expansion tank, passes down from the overflow pipe, which is shown again at E, and is led into the condenser. As at the Western Infirmary, the steam coming from the expansion tank is condensed in the condenser and passes through the pipe H to the hot well from which the boiler is fed. In addition to the above alterations, diaphragm valves, shown at O, control the proportion of steam and water forming the mixture in the motor pipe, through the valves N, which are shown in the pipe connecting the condenser with the circulator. The diaphragms of the valves O are

Diagram of the "Reck" System of Hot-water Heating as Fixed at the Royal Hospital for Sick Children, Glasgow.

A, Expansion tank; B, Circulator; C, Motor pipe; D, Main flow pipe; E, Overflow pipe; F, Condenser; G, Main return pipe; H, Condensation pipe; J, Automatic air valve; K, Automatic air valve; L, Vacuum valve; M, Steam supply pipe; N, Control valves; O, Diaphragms; P, Pressure gauge.

* Previous articles appeared on July 31 and September 11, 1915.

set to operate with whatever pressure of steam may be desired between 2½ and 7½ lb. per sq. in. The pressure and quantity of steam required will depend upon the quantity of water to be circulated and the rate of flow required. The weights shown on the diagram are arranged so that the valves N are full open when the steam pressure entering the circulator is 7½ lb. per sq. in.

An automatic air-valve is fixed to the condenser; it is shown at J on the diagram, and is arranged to keep the space above the water-line in the expansion tank open to the atmosphere, except when there is an excessive discharge of steam. It is the space above the water-line in the expansion tank that is in connection with the condenser, and through which steam and hot water overflow into the condenser, and so the air-valve attached to the condenser keeps the air space in the expansion tank open to the atmosphere. When there is an excessive discharge of steam, the air-valve closes. As at the Western Infirmary also, there is an automatic air-valve and an automatic vacuum-valve attached to the steam pipe. They are shown at K L in the right-hand diagram. They perform the same office as those at the Western Infirmary.

There are three separate Reck circulator outfits in the Children's Hospital. One is fixed in the tower of the east staircase, a second is fixed in the tower of the west staircase, and a third is fixed on the gable wall of the washhouse. The condenser is very similar to the surface condenser employed with steam-power plants. It is a cylinder carrying a number of tubes held between plates at each end. The return water from the radiators flows through the tubes, the steam from the expansion tank flowing over the outsides of the tubes, the condensate passing on to the hot well.

### The Mixing System.

The mixing system described in the previous article is also employed at the Children's Hospital. There are central mixing systems and local mixing systems, all practically arranged on the same lines, all designed to control the temperature of the water in larger or smaller groups of radiators by increasing or decreasing the flow of the hotter or colder water.

### The Boiler Plant.

At both the Children's Hospital and the Western Infirmary, Lancashire steam boilers are employed, built for pressures of 100 lb. per sq. in. The pressure is reduced to 45 lb. per sq. in. for general work, and to the lower pressures mentioned for the circulators.

### The Supply of Hot Water.

The supply of hot water for domestic purposes is provided by means of calorifiers. The large calorifiers with reservoirs are employed, and the hot-water supply is on the now usual continuous or loop system. The hot water is continually circulating from the high-temperature valve of the calorifier through the pipes from which the hot-water supply is taken, and returns to the low-temperature valve of the calorifier; branch pipes being led from the loop system to the different draw-off pipes. The calorifiers are arranged on the now also usual system by which the tubes carrying the steam can be withdrawn for cleansing purposes. Steam passes through a nest of tubes inside the cylinder, the water flowing around them, the make-up for that used being provided in the usual way. The ability to withdraw the nest of tubes is of considerable advantage, as there is sometimes a deposit from the water upon the surfaces of the tubes.

### Ventilation.

The heating of the whole of the building is carried out by hot-water radiators supplied on the system described above, except the operating block, the bathrooms, the sink-rooms, and the washrooms; all of these are heated by steam radiators. The writer proposes to deal in a separate article with the heating of operating theatres.

The radiators in the wards are supported from the walls by strong brackets and top stays fixed so that there is a space of 6 in. between the radiator and the wall and 10 in. between the radiator and the floor. The radiators have no feet. All the radiators in parts occupied by patients are fitted with metal shields covering the front and top, the upper part of the front and top being perforated, the whole being arranged to the architect's design. Fresh-air inlets are arranged behind all the radiators that are fixed against the outer walls. Each inlet is connected to a short vertical shaft in which is fixed a louvred register. The opening on the outside is fitted with an open lattice grating, and the opening into the room with a hinged cleaning-door. The register is fixed in a position where it can easily be moved and cleaned. A copper gauze filter screen is fixed diagonally across the inlet, and is arranged to slide in and out through the cleaning-door. The vitiated air is drawn out through shafts leading from the wards into fan-chambers in the roof; sirocco-centrifugal fans fixed in the room exhaust the air from the fan-chambers and force it into the outer atmosphere. The fans are driven by electric motors. The shafts through which the vitiated air is withdrawn open in the wards at the floor level, at a certain distance up on the side walls, and at the centre of the ceiling.

The heating and ventilating plant have also been fixed by Messrs. James Boyd and Co., of Paisley, at the Children's Hospital.

## Figures and Tables

**Figure f1:**